# Association Between Indole-3-Pyruvic Acid and Change in Fat-Free Mass Relative to Weight Loss in Patients Undergoing Sleeve Gastrectomy

**DOI:** 10.3390/metabo14080444

**Published:** 2024-08-11

**Authors:** Eunhye Seo, Yeongkeun Kwon, Sungsoo Park

**Affiliations:** 1 College of Nursing, Keimyung University, Daegu 42601, Republic of Korea; ehseo@kmu.ac.kr; 2 Department of Surgery, Division of Foregut Surgery, Korea University College of Medicine, Seoul 02841, Republic of Korea; kukwon@korea.ac.kr; 3 Center for Obesity and Metabolic Diseases, Korea University Anam Hospital, Seoul 02841, Republic of Korea

**Keywords:** aromatic amino acids, fat-free mass, indole-3-pyruvic acid, obesity, sleeve gastrectomy

## Abstract

Sleeve gastrectomy typically leads to weight loss, including a reduction in fat-free mass (FFM). Studies have shown significant FFM loss within 1 year after the procedure but with individual variations. This study aimed to assess whether preoperative amino acid metabolite levels can predict FFM changes following sleeve gastrectomy. This study involved 42 patients. Body weight, fat mass (FM), and FFM were measured preoperatively and 3, 6, and 12 months postoperatively. All participants experienced weight loss, FM reduction, and FFM decrease for up to 3 months after surgery. However, the following distinct groups emerged from 3 to 6 months postoperatively: one showed FFM gain relative to weight loss, whereas the other exhibited continued FFM reduction relative to weight loss. This trend persisted for up to 12 months postoperatively and became more pronounced. The group with FFM gain had lower preoperative BMI and higher levels of indole-3-pyruvic acid (IPyA). Logistic regression and ROC curve analyses confirmed IPyA’s ability to predict FFM gain between 3 and 6 months after sleeve gastrectomy, with a useful cutoff value of 20.205. Preoperative IPyA levels were associated with FFM gain relative to weight loss in the 3 to 6 months following sleeve gastrectomy. These findings suggest that IPyA may be a potential predictor for FFM changes during this period.

## 1. Introduction

Sleeve gastrectomy is currently the most widely used surgical procedure for the treatment of morbid obesity [1], providing stable weight loss, safety, and long-term resolution of obesity-related conditions, such as type 2 diabetes [2]. Sleeve gastrectomy involves the removal of a substantial portion of the gastric fundus along the greater curvature [3], leading primarily to weight loss through calorie intake restriction and a significant reduction in fat mass (FM). However, rapid weight loss following sleeve gastrectomy inevitably involves changes in fat-free mass (FFM). Although excess loss of FFM is undesirable, sleeve gastrectomy results in a greater loss of FFM than other commonly used surgical procedures, such as gastric bypass [4].

FFM consists of 30–50% skeletal muscle tissue, with the remaining portion comprising bones and other non-fat tissues. FFM plays a significant role in various metabolic mechanisms, particularly in functional abilities and bone modeling [5,6]. Thus, excess loss of FFM after sleeve gastrectomy is associated with a reduced quality of life and an increased risk of sarcopenia and osteoporosis [7]. Excess FFM loss also negatively affects the resting metabolic rate, hindering additional weight loss and increasing the risk of long-term weight regain [8,9]. From this perspective, the significance of preserving FFM during post-bariatric surgery management is widely acknowledged.

Previous studies have observed that patients can experience significant FFM loss within 1 year after bariatric surgery, with substantial interindividual variability [2]. Hence, strategies are needed to preoperatively predict patients who are at risk of excess FFM. Several researchers have reported factors influencing postoperative FFM, such as older age, male sex, and higher body mass index (BMI) [10]. However, there is still a substantial shortage of evidence in clinical settings for the prediction and management of postoperative FFM loss.

Emerging evidence suggests that gut microbiota and associated metabolites play a significant role in physiological changes, including the effects of surgery [11,12]. Among the metabolites originating from aromatic amino acids (AAAs), in particular, indole compounds, including gut microbial metabolites derived from tryptophan, can influence metabolism and energy homeostasis [13]. In our previous study, early postoperative changes in large neutral amino acids, phenylalanine profiles, and tryptophan-derived gut microbial metabolites were associated with insulin secretion and resistance [14]. Serotonin and the serotonin/5-hydroxytryptophan ratio were revealed as indicators for predicting the rate of weight loss after sleeve gastrectomy [15]. Based on previous results explaining the relationship among energy metabolism, weight loss, and metabolites [14,15], this study analyzed AAAs as a factor for predicting the initial changes in FFM following sleeve gastrectomy.

## 2. Materials and Methods

### 2.1. Study Participants

From January 2019 to December 2022, data from patients aged 20 to under 65 years who underwent sleeve gastrectomy at the Center for Obesity and Metabolic Diseases, Korea University Anam Hospital, Seoul, South Korea (Institutional Review Board approval number: 2022AN0367), were analyzed. The patients had a BMI ≥ 35 kg/m² or BMI ≥ 30 kg/m² with at least one obesity-related comorbidity. The data of patients who underwent other complex abdominal surgeries, or who suffered from uncontrolled medical or psychiatric conditions, were excluded from the analysis. Data from a total of 42 patients were included in the analysis. Results from follow-up assessments conducted before surgery and at 3, 6, and 12 months post-surgery were analyzed separately. Patient consent was waived because of the retrospective analysis of follow-up test results in this study.

### 2.2. Preoperative Education on Nutrition and Exercise Habits

Patients who underwent surgery at this center were assessed by a collaborative team of bariatric physicians, nurses, and nutritionists for dietary habits, physical activity, psychosocial factors, and weight loss goals. Dietary and exercise changes were made 3 weeks before surgery, including protein intake adjustments (0.8–1.0 g/kg), personalized dietary guidance, and moderate-intensity aerobic exercise (150–200 min, three times per week). Monitoring via phone calls or text messages ensured adherence to these changes [15].

### 2.3. Surgery and Postoperative Care

A single experienced surgeon performed sleeve gastrectomy, starting 3 cm from the gastroesophageal junction, using a 30-French bougie. This procedure reduces the stomach volume by 80–85% [14]. Following surgery, the patients adhered to a structured dietary and exercise plan. Typically, they began a low-carbohydrate clear liquid diet within 24 h of surgery, with solid meals introduced at 3–4 weeks and regular meals at 9 weeks. Their daily protein intake was set at 60 g and supplemented with amino acids, including lysine, isoleucine, valine, phenylalanine, tyrosine, and tryptophan. These supplements were administered using Promax^®^ (Korean Medical Food Co., Ltd., Seoul, Republic of Korea). During the first 4 weeks post-surgery, the patients initiated light walking exercises, gradually progressing to moderate-to high-intensity aerobic exercises for a minimum of 200 min per week and strength training for at least 20 min, 3 days per week, from weeks 5 to 26 post-surgery. To ensure compliance with the nutritional and exercise recommendations, all participants received checks and motivation every 2 weeks via phone calls or text messages [15].

### 2.4. Measurements

Pre- and postoperative visits, including weight assessments, body composition evaluations, and blood tests, were conducted according to the institution’s policy as follows: 3–7 days before surgery and at 1, 3, 6, 9, and 12 months after surgery, provided that there were no specific medical concerns related to health conditions. Anthropometric measurements were performed at each visit. Body composition was determined using bioimpedance analysis (BIA) with the Quad Scan 4000 multi-frequency bioelectrical impedance analyzer from BodyStat^®^, U.K. FM and FFM, which include skeletal muscle, bones, and lean soft tissue, were calculated by measuring impedance at 50 kHz and applying the BodyStat^®^ equation. The standard error for BIA is approximately 3.5% (kg m^−2^).

Serum samples for measuring AAAs were collected after the patients completed a 3-week preoperative education program on nutrition and exercise habits. The patients fasted for 8 h before sampling [16]. Metabolite profiling was performed using liquid chromatography (Vanquish UHPLC, Thermo Fisher Scientific, Waltham, MA, USA) coupled with mass spectrometry (QE Orbitrap MS, Thermo Fisher Scientific, Waltham, MA, USA). Serum samples were prepared by adding 5 μL of internal standard to 200 μL of serum, followed by protein precipitation with 700 μL of cold methanol and centrifugation. The supernatant was then dried, re-dissolved in 100 μL of 0.1% formic acid in water, and analyzed. Chromatographic separation was achieved using a Waters ACQUITY UPLC^®^ HSS T3 column with a gradient at a flow rate of 0.4 mL/min and a temperature of 35 °C. Mass spectrometry was conducted in Parallel Reaction Monitoring mode with a resolution of 35,000, utilizing heated electrospray ionization in positive mode [14,15].

### 2.5. Outcome Measures

The main outcome of this study was the ratio of relative FFM change to weight loss. The metric was calculated using the following formula: FFM loss/weight loss % = FFM (post) − FFM (pre)/weight (post) − weight (pre) × 100 [4,10]. All participants included in this study experienced weight loss between 3 and 6 months postoperatively. Thus, FFM gain was defined when the calculated value of the FFML/weight loss was negative, whereas FFM loss was defined when this value was positive.

### 2.6. Statistical Analyses

Data are presented as means ± standard deviation. Data were analyzed using SPSS 23.0 software (IBM SPSS, Inc., Chicago, IL, USA). The normality of the variables was confirmed using the Shapiro–Wilk test, and based on the results, group comparisons were conducted using the independent Student’s t-test or Mann–Whitney U test. Categorical variables were assessed using the chi-squared test. Statistical significance was determined at a 5% alpha level (95% confidence interval) with 80% power. A *p*-value < 0.05 was considered statistically significant. Logistic regression models were generated to investigate the associations between serum aromatic amino acid-derived metabolites and post-3 to -6 month FFM changes after adjusting for age, sex, baseline BMI, weight, FM, and FFM. The receiver operating characteristic (ROC) curve and area under the curve (AUC) were used to determine the discriminatory power of the metabolites on FFM gain.

## 3. Results

### 3.1. Baseline Characteristics

This study included 42 participants with an average age of 39.93 ± 10.91 years, of which 27 were female, accounting for 64.2% of the total. The mean BMI was 40.85 ± 6.53 kg/m², and the average body weight was 114.97 ± 23.40 kg. The mean FM and FFM were 55.35 ± 15.22 kg and 32.03 ± 5.73 kg, respectively (Table 1).

### 3.2. Predictors for FFM Gain Relative to Weight Loss between 3 and 6 Months after Sleeve Gastrectomy

The anthropometric parameters and metabolites at baseline for the FFM gain and loss groups between 3 and 6 months are shown in Table 2. At baseline, the variables that showed significant differences between the FFM gain group and the FFM loss group were BMI (*p* = 0.048) and indole-3 pyruvic acid (IPyA) (*p* = 0.007). Unadjusted outcomes for FFM gain prediction by BMI (OR = 0.080; 95% CI, 0.770–1.015) and IPyA (OR = 1.123; 95% CI, 1.023–1.233) are presented in Model 1. In Model 2, adjustments were made for age, sex, weight, FM, and FFM, resulting in higher odds of IPyA predicting FFM gain (OR, 1.217; 95% CI, 1.019–1.454) (Table 3).

**Figure 1:**
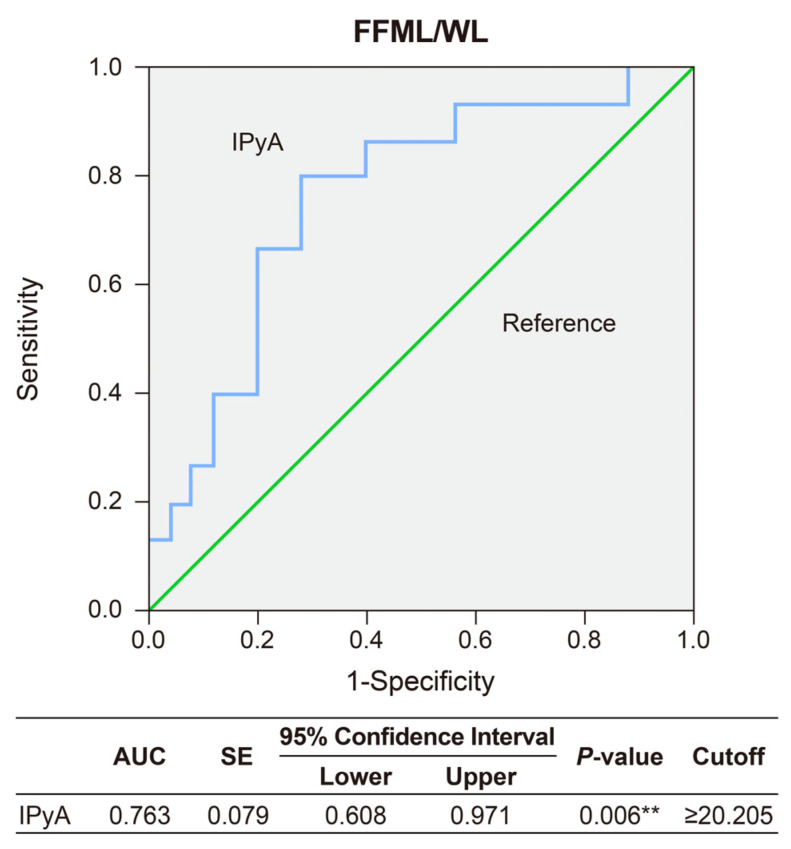
ROC curve analysis of preoperative IPyA values for predicting FFM gain between 3 and 6 months post-surgery. ** *p* < 0.01. Abbreviation: AUC, the area under the ROC curve; BMI, body mass index; FFM, fat-free mass; FM, fat mass; IPyA, indole-3 pyruvic acid; ROC, receiver operating characteristic; WL, weight loss.

ROC curves were generated using IPyA, which was significantly associated with FFM gain between 3 and 6 months after sleeve gastrectomy. IPyA exhibited superior performance in predicting FFM gain. The value of the AUC on IPyA was 0.763 (*p* = 0.006; 95% CI 0.608–0.971,). For FFM gain, the IPyA cutoff was 20.205 µmol/L and associated with 80% sensitivity and 40% specificity (Figure 1).

### 3.3. Body Composition and Weight Changes Up to 1 Year After Surgery between Groups Based on the Relative FFM Changes to Weight Loss

**Figure 2:**
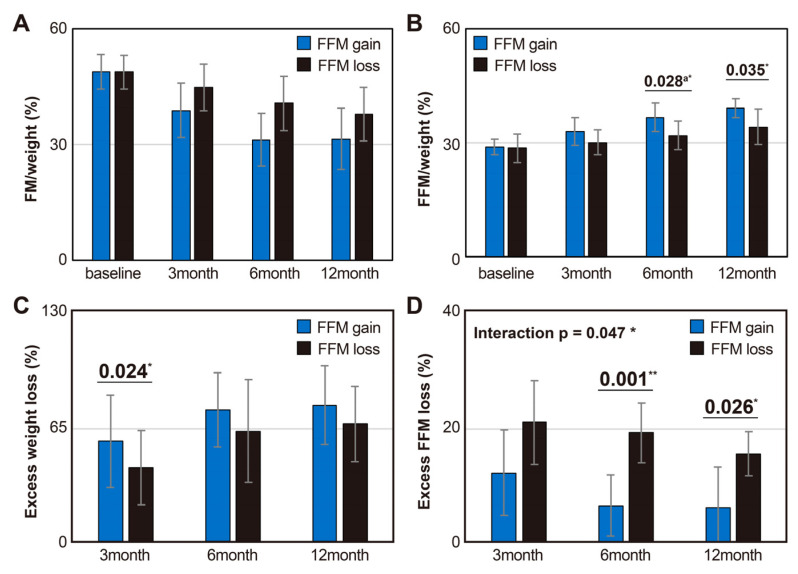
Body composition and weight changes up to 1 year after surgery between groups based on FFM change relative to weight loss. (**A**) The ratio of FM to total body weight, (**B**) The ratio of FFM to total body weight, (**C**) Percentage reduction in excess weight based on BMI 25 after surgery, (**D**) Percentage of excess FFM loss exceeding 25% after surgery. Values are presented as means ± standard deviations or numbers (%). The *p*-value was calculated using Student’s *t*-test. The ^a^ *p*-value was calculated using the Mann–Whitney U test. * *p* < 0.05, ** *p* < 0.01. Abbreviation: FFM, fat-free mass; FM, fat mass.

Over a period of baseline to 12 months, both the FFM gain and FFM loss groups exhibited a decrease in body weight relative to FM percentage (FM/weight) (interaction between group and time, *p* = 0.595) (Figure 2A), whereas the body weight-relative FFM percentage (FFM/weight) gradually increased (interaction between group and time, *p* = 0.584). FFM/weight was significantly higher in the FFM gain group than in the FFM loss group at 6 (*p* = 0.028) and 12 months (*p* = 0.035) post-surgery (Figure 2B).

The excess weight loss percentage increased over time, with the FFM gain group measuring 63.15 ± 26.88%, 76.94 ± 21.50%, and 75.64 ± 22.83% and the FFM loss group measuring 42.62 ± 21.63%, 63.79 ± 29.68%, and 68.05 ± 21.72% at 3, 6, and 12 months post-surgery. The excess weight loss percentage was significantly higher in the FFM gain group than in the FFM loss group at 3 months post-surgery (*p* = 0.024), and this trend persisted at 6 and 12 months post-surgery (Figure 2C). The total weight loss percentage also increased gradually, with the FFM gain group measuring 21.39 ± 2.94%, 27.88 ± 2.85%, and 30.08 ± 3.49% and the FFM loss group measuring 16.54 ± 1.22%, 24.29 ± 1.89%, and 28.39 ± 1.94%, at 3, 6, and 12 months post-surgery, respectively. The excess weight loss percentage increased over time, with the FFM gain group measuring 63.15 ± 26.88%, 76.94 ± 21.50%, and 75.64 ± 22.83% and the FFM loss group measuring 42.62 ± 21.63%, 63.79 ± 29.68%, and 68.05 ± 21.72% at 3, 6, and 12 months post-surgery. The excess weight loss percentage was significantly higher in the FFM gain group than in the FFM loss group at 3 months post-surgery (*p* = 0.024), and this trend persisted at 6 and 12 months post-surgery (Figure 2C). The total weight loss percentage also increased gradually, with the FFM gain group measuring 21.39 ± 2.94%, 27.88 ± 2.85%, and 30.08 ± 3.49% and the FFM loss group measuring 16.54 ± 1.22%, 24.29 ± 1.89%, and 28.39 ± 1.94%, at 3, 6, and 12 months post-surgery, respectively.

Excess FFM loss, which was quantified as the reduced FFM amount relative to the reduced body weight, exhibited a significant interaction over time and between groups (*p* = 0.047). At 6 (*p* = 0.001) and 12 months post-surgery (*p* = 0.026), the excess FFM loss ratio in the FFM gain group was significantly lower than that in the FFM loss group (Figure 2). The raw data for weight, FM, and FFM changes at baseline, as well as at 3, 6, and 12 months post-surgery, categorized into overall and FFM loss and FFM gain groups, are detailed in Supplementary Table S1.

## 4. Discussion

In this study, we demonstrated an association among the preoperative IPyA profile, a gut microbiota-derived metabolite, and changes in FFM relative to weight loss from 3 to 6 months post-surgery. This finding suggests that the preoperative IPyA profile could serve as a biomarker to predict the initial changes in FFM relative to weight loss after sleeve gastrectomy. This finding has the potential to enhance our understanding of patient responses to surgery and facilitate the development of tailored treatment approaches for individual patients.

The primary goal of modern obesity treatment is to optimize weight loss while preserving metabolically active FFM [17]. Patients undergoing sleeve gastrectomy have been reported to experience a significant amount of FFM loss, exceeding 8 kg within the first year after surgery [18,19]. Observing the progression of FFM loss over 3 years, it was noted that substantial FFM loss occurred at 3 and 6 months post-surgery, accounting for 57% and 73% of the total loss, respectively [10], emphasizing the need to implement interventions aimed at mitigating such FFM losses either preoperatively or during the early stages of postoperative recovery [19]. However, clinical data on postoperative FFM changes are limited, and the methods for measuring FFM changes vary among studies.

Barzin et al. analyzed the prevalence of excess FFM loss following sleeve gastrectomy, utilizing different cutoff points of 25%, 30%, and 35%. Their study found that 1 month after surgery, 80–90% of patients experienced excess FFM loss. This percentage then decreased to 60–20% at 6 months post-surgery and increased once more to 60–40% at 36 months post-surgery [4]. Nuijten et al., utilizing excess FFM loss calculated in the same manner, demonstrated that excess FFM loss decreased up to 6 months after surgery, but began increasing at 12 months, exceeding the level observed at 3 months by 36 months. Among the 574 participants, 16% underwent sleeve gastrectomy and 84% received RYGB [10]. Patients experiencing weight regain 2 years after surgery were found to have lower FFM ratios and resting energy expenditure (REE) relative to body weight compared with those maintaining stable body weight [20]. The results of these studies suggest that the increase in weight regain observed following sleeve gastrectomy may be influenced by excess loss of FFM, which affects metabolism, thermoregulation, functional capacity, and weight recovery [21,22]. Furthermore, these findings indicate that the preservation of FFM may play a role in limiting the reduction in REE, a key criterion for long-term weight regain [23]. Therefore, during the period of rapid changes in weight and body composition following sleeve gastrectomy, an increase in FFM relative to weight loss can be predictive of positive clinical outcomes. Indeed, in our study, when an increase in FFM relative to weight loss occurred between 3 and 6 months after surgery, we observed a significant reduction in excess FFM loss accompanied by stable weight loss at 6 and 12 months after surgery (Figure 2).

Our results emphasizing the IPyA metabolite in the indole pathway of tryptophan to predict an increase in FFM relative to weight after sleeve gastrectomy can be considered in the context of previous research on the effects of skeletal muscle, which constitutes approximately 40% of FFM [19]. The IPyA pathway represents one of the routes of tryptophan metabolism by the gut microbiota, offering indole derivatives capable of activating the aryl hydrocarbon receptor (AhR) signaling pathway [24] and enabling the modulation of intestinal immune responses [25]. Based on the AhR activation mechanism, IPyA plays a potential role as a preventative factor in modulating inflammatory responses in animal models of chronic colitis and rheumatoid arthritis [26,27]. However, interleukin-4-induced-1 (IL4I1), an essential enzyme catalyzing indole metabolism, is highly expressed in muscle stem cells (MuSCs) that are exposed to inflammatory cytokines [28]. In other words, in MuSCs activated by inflammation due to the impact of adipose tissue toxicity, IPyA generated by IL4I1 can play a ligand role in activating AhR [29], potentially mediating neutrophil infiltration inhibition and reducing reactive oxygen species levels [28].

MuSCs, also known as satellite cells, are responsible for tissue repair through self-regeneration and muscle differentiation [30]. Clinical studies on the treatment of skeletal muscle disorders are being actively conducted based on the myogenic capabilities of MuSCs [31,32]. MuSCs have a bidirectional role, serving as key regulators of inflammation resolution by releasing various paracrine factors while simultaneously undergoing proliferation and differentiation in response to signals from pro-inflammatory mediators [33]. In other words, IPyA-activated AhR bidirectionally contributes to both anti-inflammatory actions and muscle tissue regeneration by MuSCs. Therefore, in our study, preoperative serum IPyA levels may have influenced postoperative changes in muscle regeneration and differentiation.

Our study had several limitations. Changes in postoperative body composition can be influenced by factors such as calorie intake and expenditure as well as the consumption of amino acid supplements. Although we monitored compliance with recommendations for weekly meals, exercise, protein supplement use, indirect supervision through phone calls and text messages may have introduced confounding factors. The retrospective nature of our study introduces inherent limitations related to data selection and potential confounding variables. Furthermore, we acknowledge that the small sample size of 42 patients may limit the statistical power of our study. This restriction affects the ability to detect statistically significant associations and may influence the generalizability of our findings. Therefore, follow-up studies are necessary to validate our results and improve the accuracy of predictions regarding body composition changes post-surgery.

## 5. Conclusions

In summary, it was observed that preoperative AAAs level, especially IPyA levels exceeding 20.205 µmol/L, were associated with an anticipated increase in FFM relative to weight loss during the 3 to 6 months following sleeve gastrectomy. This effect may be primarily attributed to the influence of IPyA on inflammation, regeneration, and differentiation within the skeletal muscles of FFM. Additionally, an increase in FFM relative to weight loss may contribute to long-term effects by mitigating the risk of weight regain that is associated with reduced REE following sleeve gastrectomy. Further research is needed to determine whether measuring preoperative serum AAAs can be of long-term benefit for FFM gain and to understand the biological mechanisms by which IPyA contributes to FFM gain.

## Figures and Tables

**Table 1:** Baseline characteristics.

Variables	Baseline (*n* = 42)
Age, years	39.93 ± 10.91
Female sex, no. (%)	27 (64.2)
BMI, kg/m^2^	40.85 ± 6.53
Weight, kg	114.97 ± 23.40
FM/weight, %	48.80 ± 4.28
FFM/weight, %	32.03 ± 5.67

Values are presented as mean ± standard deviation or number (%). Abbreviation: BMI, body mass index; FFM, fat-free mass; FM, fat mass.

**Table 2:** Anthropometric parameters and metabolites at baseline in FFM gain and FFM loss between 3 and 6 months.

	Baseline		
	FFM Gain (*n* = 16)	FFM Loss (*n* = 26)	*p*-Value
ΔFFM/ΔW	–15.59 ± 11.65	18.83 ± 17.76	-
Age, years	42.50 ± 9.85	38.21 ± 11.43	0.228
Female sex, no. (%)	9 (56.2)	18 (69.2)	0.406
Anthropometric parameters			
BMI, kg/m^2^	38.39 ± 5.16	42.27 ± 6.89	0.048 *
Weight, kg	111.51 ± 21.39	116.97 ± 24.67	0.478
FM/weight, %	45.58 ± 7.16	48.80 ± 4.38	0.220
FFM/weight, %	30.44 ± 6.38	33.00 ± 5.19	0.992
Serum analysis			
AST, U/L	39.40 ± 16.02	47.80 ± 23.24	0.117
ALT, U/L	59.80 ± 27.04	65.30 ± 30.88	0.635
BUN, mg/dL	14.52 ± 5.18	14.23 ± 5.59	0.893
Creatinine, mg/dL	0.90 ± 0.37	0.84 ± 0.24	0.707
Bilirubin, mg/dL	0.71 ± 0.44	0.75 ± 0.31	0.472 ^a^
Uric acid, mg/dL	5.99 ± 1.96	5.91 ± 1.68	0.922
Fasting blood glucose, mg/dL	144.10 ± 44.01	136.11 ± 42.68	0.644
Cholesterol, mg/dL	197.80 ± 35.37	177.62 ± 38.04	0.164
HDL, mg/dL	56.00 ± 21.61	41.57 ± 5.57	0.066
LDL, mg/dL	134.00 ± 42.72	111.57 ± 33.21	0.165
TG, mg/dL	178.78 ± 102.75	169.90 ± 86.52	0.824
Aromatic amino acids, µmol/L			
Tryptophan	57.02 ± 11.69	54.05 ± 13.43	0.469
IPyA	27.62 ± 10.43	18.31 ± 8.52	0.007 **
IAA	1.68 ± 0.85	1.49 ± 1.03	0.133 ^a^
ILA	0.69 ± 0.14	0.62 ± 0.19	0.236
IPA	1.35 ± 2.92	0.46 ± 0.55	0.138
5-HTP	0.21 ± 0.76	0.08 ± 0.30	0.428
5-HT	0.24 ± 0.21	0.26 ± 0.24	0.834
5-HIAA	0.04 ± 0.01	0.04 ± 0.01	0.170 ^a^
Phenylalanine	67.12 ± 9.63	67.85 ± 12.40	0.841
Tyrosine	56.19 ± 16.61	58.94 ± 15.91	0.595
L-DOPA	0.58 ± 0.37	0.48 ± 0.21	0.247
Dopamine	0.11 ± 0.08	0.10 ± 0.07	0.506

Values are presented as means ± standard deviation or number (%). The *p*-value was calculated using Student’s t-test. The ^a^ *p*-value was calculated using the Mann–Whitney U test. * *p* < 0.05, ** *p* < 0.01. Abbreviations: 5-HT, 5-hydroxytryptamine; 5-HTP, 5-hydroxytryptophan; 5-HIAA, 5-hydroxyindole acetic acid; ALT, alanine aminotransferase; AST, aspartate aminotransferase; BMI, body mass index; BUN, blood urea nitrogen; FFM, fat-free mass; FM, fat mass; IAA, indole-3-acetic acid; ILA, indole-3-lactic acid; IPA, indole-3-propionic acid; IPyA, indole-3 pyruvic acid; L-DOPA, L-3,4-dihydroxyphenylalanine.

**Table 3:** Odds ratio estimates and 95% confidence intervals from logistic regression models for FFM gain relative to weight loss between 3 and 6 months after surgery.

**Model 1**				
**Step 1**	***B* (s.e)**	***p*-Value**	**Odds Ratio**	**95% CI**
BMI	−0.123 (0.070)	0.080	0.080	0.770–1.015
IPyA	0.116 (0.048)	0.015 *	1.123	1.023–1.233
R^2^ = 0.357, F = 8.00, *p* = 0.002 **				
**Model 2**				
**Step 1**	***B* (s.e)**	***p*-value**	**Odds Ratio**	**95% CI**
Age	−0.054 (0.090)	0.550	0.948	0.794–1.130
Sex	1.216 (1.893)	0.521	3.374	0.083–137.926
BMI	0.646 (0.435)	0.138	1.908	0.813–4.476
Weight	0.470 (0.306)	0.125	1.600	0.878–2.913
FM	−0. 799 (0.377)	0.034 *	0.450	0.215–0.941
FFM	−0. 892 (0.639)	0.163	0.410	0.117–1.433
IPyA	0.217 (0.106)	0.041 *	1.242	1.009–1.529
**Step 4**	***B* (s.e)**	***p*-value**	**Odds Ratio**	**95% CI**
BMI	0.534 (0.354)	0.131	1.706	0.853–3.412
FM	−0.307 (0.178)	0.084	0.736	0.519–1.042
IPyA	1.217 (0.091)	0.030 *	1.217	1.019–1.454
R^2^ = 0.576, F = 8.00, *p* = 0.001 **				

Values are presented as means ± standard deviation or numbers (%). * *p* < 0.05, ** *p* < 0.01. Abbreviations: BMI, body mass index; FFM, fat-free mass; FM, fat mass; IPyA, indole-3 pyruvic acid.

## Data Availability

Data are available upon reasonable request from the corresponding author.

## Supplementary Materials

The following supporting information can be downloaded at https://www.mdpi.com/article/10.3390/metabo14080444/s1. Table S1: The changes in weight, FM, FFM changes at baseline, and at 3, 6, and 12 months post-surgery.
