# Supplementary material for: Association Between Indole-3-Pyruvic Acid and Change in Fat-Free Mass Relative to Weight Loss in Patients Undergoing Sleeve Gastrectomy
Source: Metabolites. 2024 Aug 11;14(8):444. doi: 10.3390/metabo14080444 (PMC11356315; doi:10.3390/metabo14080444)
Supplement: Supplementary file 1 [file metabolites-14-00444-s001.zip › metabolites-3113145-supplementary.pdf]

Supplementary Table S1. The changes in weight, FM, FFM changes at baseline, and at 3, 6, and 12 months post-surgery

	Total (*n* = *42*)				FFM gain (*n* = 16)				FFM loss (*n* = *26*)			
	Baseline	3 mo	6 mo	12 mo	Baseline	3 mo	6 mo	12 mo	Baseline	3 mo	6 mo	12 mo
Weight, kg	114.97 ± 23.40	93.03 ± 18.29	84.82 ± 16.89	84.28 ± 11.85	111.51 ± 21.39	85.83 ± 11.27	79.30 ± 10.05	79.21 ± 9.03	116.97 ± 24.67	97.47 ± 20.79	88.22 ± 19.68	87.76 ± 12.91
BMI	40.85 ± 6.53	33.88 ± 5.68	30.43 ± 4.99	29.65 ± 4.53	38.39 ± 5.16	31.49 ± 3.87	28.86 ± 3.20	28.48 ± 3.30	42.27 ± 6.89	35.30 ± 6.27	31.52 ± 5.85	30.33 ± 5.18
Total weight loss, %	-	18.06 ± 8.64	25.16 ± 9.90	28.23 ± 10.88	-	21.39 ± 11.78	27.88 ± 11.41	30.08 ± 14.00	-	16.54 ± 6.12	24.29 ± 9.50	28.39 ± 9.71
Excess weight loss, %	-	48.48 ± 4.20	68.69 ± 4.45	70.63 ± 3.32	-	57.99 ± 4.85	76.04 ± 3.55	78.87 ± 2.81	-	42.62 ± 3.61	63.79 ± 4.95	68.05 ± 3.62
FM/weight, %	48.80 ± 4.28	42.55 ± 6.89	36.97 ± 8.27	35.29 ± 7.83	45.58 ± 7.16	38.83 ± 7.02	31.21 ± 6.90	31.42 ± 7.94	48.80 ± 4.38	44.78 ± 6.04	40.73 ± 7.10	37.93 ± 6.98
FFM/weight %	32.03 ± 5.67	28.83 ± 5.12	28.56 ± 5.28	28.80 ± 5.59	30.44 ± 6.38	28.28 ± 4.47	29.11 ± 4.61	29.33 ± 5.12	33.00 ± 5.19	29.17 ± 5.64	28.23 ± 5.81	27.23 ± 4.98
Excess FFM loss, %	-	17.80 ± 15.35	14.03 ± 12.29	11.99 ± 11.27	-	12.23 ± 7.65	6.40 ± 5.51	6.04 ± 7.13	-	21.23 ± 7.47	19.33 ± 5.33	15.63 ± 3.87

Values are presented as mean ± standard deviation or number (%). Abbreviation: BMI, body mass index; FFM, fat-free mass; FM, fat mass.
